# Phosphorylation of HIV Tat by PKR increases interaction with TAR RNA and enhances transcription

**DOI:** 10.1186/1743-422X-2-17

**Published:** 2005-02-28

**Authors:** Liliana Endo-Munoz, Tammra Warby, David Harrich, Nigel AJ McMillan

**Affiliations:** 1 Centre for Immunology and Cancer Research, University of Queensland, Princess Alexandra Hospital, Brisbane, Australia; 2 Queensland Institute of Medical Research, Royal Brisbane Hospital, Brisbane, Australia

## Abstract

**Background:**

The interferon (IFN)-induced, dsRNA-dependent serine/threonine protein kinase, PKR, plays a key regulatory role in the IFN-mediated anti-viral response by blocking translation in the infected cell by phosphorylating the alpha subunit of elongation factor 2 (eIF2). The human immunodeficiency virus type 1 (HIV-1) evades the anti-viral IFN response through the binding of one of its major transcriptional regulatory proteins, Tat, to PKR. HIV-1 Tat acts as a substrate homologue for the enzyme, competing with eIF2α, and inhibiting the translational block. It has been shown that during the interaction with PKR, Tat becomes phosphorylated at three residues: serine 62, threonine 64 and serine 68. We have investigated the effect of this phosphorylation on the function of Tat in viral transcription. HIV-1 Tat activates transcription elongation by first binding to TAR RNA, a stem-loop structure found at the 5' end of all viral transcripts. Our results showed faster, greater and stronger binding of Tat to TAR RNA after phosphorylation by PKR.

**Results:**

We have investigated the effect of phosphorylation on Tat-mediated transactivation. Our results showed faster, greater and stronger binding of Tat to TAR RNA after phosphorylation by PKR. *In vitro *phosphorylation experiments with a series of bacterial expression constructs carrying the wild-type *tat *gene or mutants of the gene with alanine substitutions at one, two, or all three of the serine/threonine PKR phosphorylation sites, showed that these were subject to different levels of phosphorylation by PKR and displayed distinct kinetic behaviour. These results also suggested a cooperative role for the phosphorylation of S68 in conjunction with S62 and T64. We examined the effect of phosphorylation on Tat-mediated transactivation of the HIV-1 LTR *in vivo *with a series of analogous mammalian expression constructs. Co-transfection experiments showed a gradual reduction in transactivation as the number of mutated phosphorylation sites increased, and a 4-fold decrease in LTR transactivation with the Tat triple mutant that could not be phosphorylated by PKR. Furthermore, the transfection data also suggested that the presence of S68 is necessary for optimal Tat-mediated transactivation.

**Conclusion:**

These results support the hypothesis that phosphorylation of Tat may be important for its function in HIV-1 LTR transactivation.

## Background

Since its isolation in 1983 [1,2], human immunodeficiency virus type 1 (HIV-1) continues to cause 5 million new infections each year, and since the beginning of the epidemic, 31 million people have died as a result of HIV/AIDS [3]. One of the major mechanisms employed by the immune system to counteract the effects of viral infections is through an antiviral cytokine – type 1 interferon (IFN). However, while IFN is able to inhibit HIV-1 infection *in vitro *[4], it has not been effective in the treatment of HIV-1 infections *in vivo*. Furthermore, the presence of increasing levels of IFN in the serum of AIDS patients while viral replication continues and the disease progresses [5-7] indicates that HIV-1 must employ a mechanism to evade the antiviral effects of IFN.

In response to viral infection, IFN induces a number of genes including the dsRNA-dependent protein kinase R (PKR). PKR exerts its anti-viral activity by phosphorylating the alpha subunit of translation initiation factor 2 (eIF2α), which results in the shut-down of protein synthesis in the cell [8]. The importance of PKR in the host antiviral response is suggested by the fact that most viruses including vaccinia [9], adenovirus [10], reovirus [11], Epstein-Barr virus [12], poliovirus [13], influenza [14], hepatitis C [15,16], human herpes virus [17-19], and SV40 [20], employ various mechanisms to inhibit its activity. HIV-1 is no exception and we and others have shown that PKR activity is inhibited by HIV via the major regulatory protein, Tat [21-23]. Productive infection by HIV-1 results in a significant decrease in the amounts of PKR [23] and HIV-1 Tat protein has been shown to act as a substrate homologue of eIF2α, preventing the phosphorylation of this factor and allowing protein synthesis and viral replication to proceed in the cell [21,22]. During the interaction between Tat and PKR the activity of the enzyme is blocked by Tat and Tat itself is phosphorylated by PKR [21] at serine 62, threonine 64 and serine 68 [22].

HIV-1 Tat is a 14 kDa viral protein involved in the regulation of HIV-1 transcriptional elongation [24-26] and in its presence, viral replication increases by greater than 100-fold [27,28]. It functions to trigger efficient RNA chain elongation by binding to TAR RNA, which forms the initial portion of the HIV-1 transcript [29]. The interaction between Tat and TAR is critical for virus replication and mutations in Tat that alter the RNA-binding site result in defective viruses. Furthermore, virus replication can be strongly inhibited by the overexpression of TAR RNA sequences that act as competitive inhibitors of regulatory protein binding [30].

While a number of reports have shown that PKR and Tat protein interact, and furthermore, that Tat is phosphorylated by PKR, none have yet addressed the issue of the functional consequences for the phosphorylation of the Tat protein. Here we examine the phosphorylation of Tat by PKR and its effect on TAR RNA binding and HIV-1 transcription, and show that the phosphorylation of Tat results in Tat protein binding more strongly to TAR RNA. Removal of the residues reported to be phosphorylated by PKR resulted in decreased Tat phosphorylation and a significant loss of Tat-mediated transcriptional activity.

## Results

### The phosphorylation of HIV-1 Tat by PKR increases its interaction with TAR RNA

**Figure 1:**
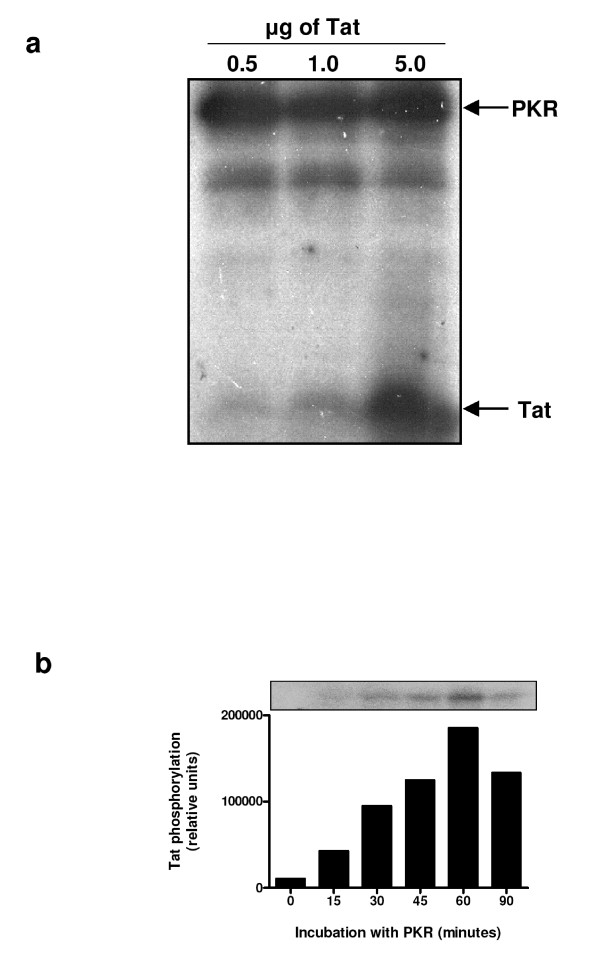
**Phosphorylation of HIV-1 Tat86 by PKR. (a) **PKR was immunoprecipitated from HeLa cell extracts and activated with synthetic dsRNA in the presence of γ-^32^P-ATP. This activated ^32^P-PKR was used to phosphorylate 0.5, 1 and 5 μg of synthetic Tat86 in the presence of γ-^32^P-ATP, at 30°C for 15 minutes. Proteins were separated by 15% SDS-PAGE. **(b) **PKR immunoprecipitated from HeLa cell extracts, and activated with dsRNA and ATP, was used to phosphorylate 2 μg of synthetic Tat86 at 30°C for the times indicated.

We first confirmed the capability of our PKR preparation immunoprecipitated from HeLa cells to phosphorylate synthetic Tat protein (aa 1–86) (Figure 1a), and we determined the optimal phosphorylation time of Tat by PKR as 60 minutes (Figure 1b). We also confirmed that Tat was not phosphorylated by PKR in the absence of ATP, or by ATP alone (data not shown).

**Figure 2:**
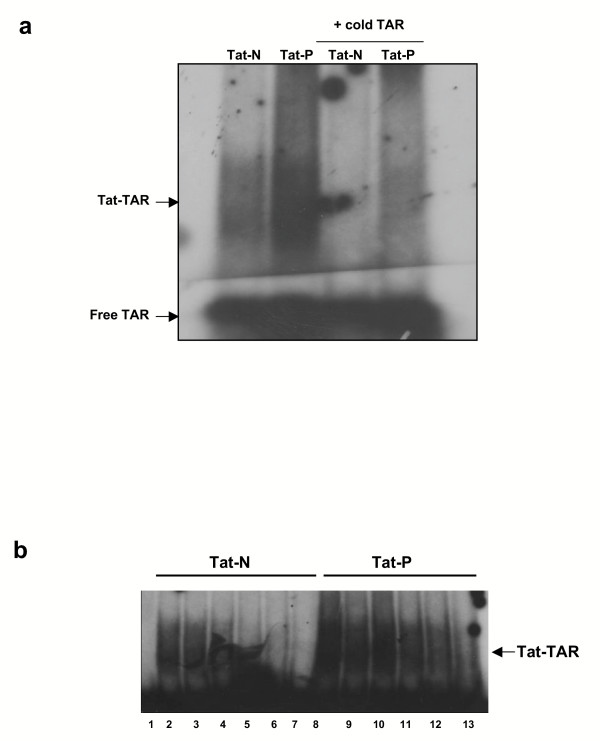
**EMSA of Tat-N, Tat-P and TAR RNA showing dissociation of the Tat-TAR complex with increasing salt concentration. (a) **PKR immunoprecipitated from HeLa cell extracts, and activated with dsRNA and ATP, was used to phosphorylate 2 μg of synthetic Tat86 at 30°C for 1 h, in the presence (Tat-P) or absence (Tat-N) of γ-^32^P-ATP. TAR RNA was synthesized *in vitro *from pTZ18TAR80 using a commercial kit, and either γ-^32^P-dCTP or unlabelled dCTP. The Tat-TAR RNA binding reaction was allowed to proceed in binding buffer at 30°C for 10 minutes. Each reaction contained 200 ng of either Tat-N or Tat-P, and approximately 70 000 cpm of ^32^P-TAR RNA (lanes 1 and 2), or approximately 70 000 cpm of ^32^P-TAR RNA and 7.5 × the volume of unlabelled TAR RNA (lanes 3 and 4). The Tat-TAR complexes formed were resolved on a 5% acrylamide/0.25X TBE gel. **(b) **The Tat-TAR binding reactions were performed at 30°C for 10 minutes in binding buffer containing various concentrations of NaCl: 25 mM (lanes 2 and 8), 50 mM (lanes 3 and 9), 100 mM (lanes 4 and 10), 200 mM (lanes 5 and 11), 500 mM (lanes 6 and 12), and 1000 mM (lanes 7 and 13). Lanes 2–7 show the dissociation of the Tat-N-TAR complex, and lanes 8–13 show the dissociation of the Tat-P-TAR complex. Lane 1 is TAR RNA only.

To address the issue of the consequences of PKR phosphorylation on Tat function we investigated the ability of phosphorylated Tat (herein called Tat-P) and normal Tat (Tat-N) to bind to HIV-1 TAR RNA. Synthetic Tat protein (aa 1–86) was phosphorylated *in vitro *using PKR previously immunoprecipitated from HeLa cells. An electrophoretic mobility shift assay (EMSA) was performed to observe any difference in the binding of Tat-N and Tat-P to TAR RNA (Figure 2a). It can be seen that Tat-N was able to form a specific Tat-TAR complex that could be effectively competed off using a 7.5-fold excess of cold TAR RNA. Tat-P was also able to form a specific Tat-TAR complex that clearly contained more TAR RNA than non-phosphorylated Tat. This complex could also be competed off using cold TAR but some residual complex was left suggesting that the Tat-P-TAR complex was more resistant to competition with cold TAR than the Tat-N-TAR complex.

As Tat-P appeared to bind more readily to TAR, we next investigated the differences in the binding efficiency of Tat-N and Tat-P with TAR RNA. EMSA were performed in the presence of increasing concentrations of NaCl (from 25–1000 mM). The progressive dissociation of the Tat-N-TAR RNA complex with increasing concentrations of salt in the buffer was observed (Figure 2b, lanes 2–7) while Tat-P-TAR complexes under the same conditions were clearly more stable (lanes 8–13). For example, at 500 mM NaCl the Tat-N-TAR complex was almost completely dissociated (lane 6) while the Tat-P-TAR complex was still clearly observed (lane 12). Even at the maximum salt concentration (1000 mM), the Tat-P-TAR complex can still be seen (lane 13), while the Tat-N-TAR complex was completely dissociated. These results suggest that Tat86 phosphorylated by PKR binds TAR RNA more efficiently and more strongly than normal Tat.

### Efficient phosphorylation of Tat requires particular residues

**Figure 3:**
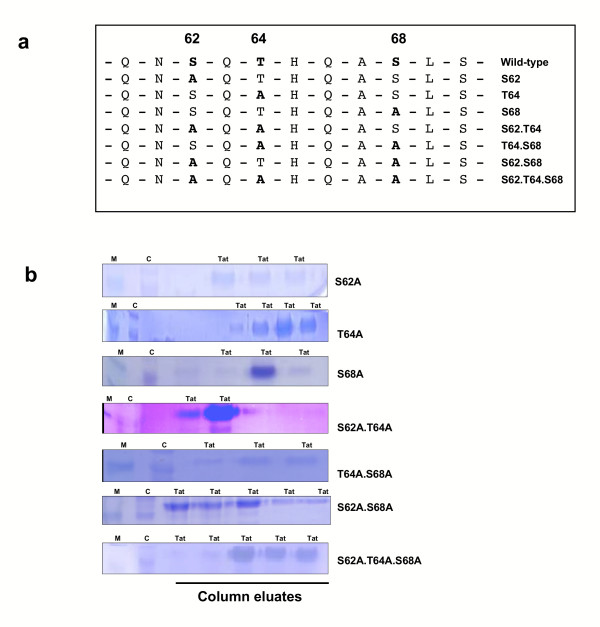
**Construction of HIV-1 Tat phosphorylation mutants. (a) **Amino acid sequence of HIV-1 Tat wild-type and mutants. Changes to alanine at serine 62, threonine 64 and serine 68 are indicated for each mutant, and compared to the wild-type protein. Mutations were introduced by site-directed mutagenesis into pET-DEST42-HIS-Tat86. **(b) **Competent BL21(DE3)pLysS cells, transformed with pET-DEST42-HIS-Tat86 wild-type or mutants, were grown and lysed with 6 M guanidine-HCl, pH 8.0. The suspension was cleaned of cell debris and loaded onto a packed metal affinity resin. The resin was washed and the HIS-tagged Tat proteins were eluted with 6 M guanidine-HCl, pH 4.0. The fractions collected were dialysed in 0.1 mM DTT and then analysed by 15% SDS-PAGE and stained with Coomassie blue. Tat lanes show fractions containing HIS-tagged Tat proteins; M lanes, 14 kDa marker; C lanes, BL21(DE3)pLysS cell extract.

Brand *et al*. [22] reported that PKR was able to phosphorylate Tat at amino acids serine-62, threonine-64 and serine-68. We therefore wished to know if any of these residues were critically important in the ability of Tat to bind TAR RNA. To this end, we created a series of Tat proteins containing mutations of all possible combinations of S62, T64 and T68 and investigated the phosphorylation of the resulting mutant Tat protein. A series of seven Tat mutants were made using alanine scanning (Figure 3a) and cloned into the bacterial expression vector pET-DEST42, which contains a C-terminal 6 × His tag to allow purification using metal affinity chromatography. The resulting constructs were validated by sequencing before the mutant Tat proteins were expressed and purified (Figure 3b). Protein yields varied between 40–170 g/mL and all mutants were full length, as confirmed by western blotting using an anti-His antibody (data not shown).

**Figure 4:**
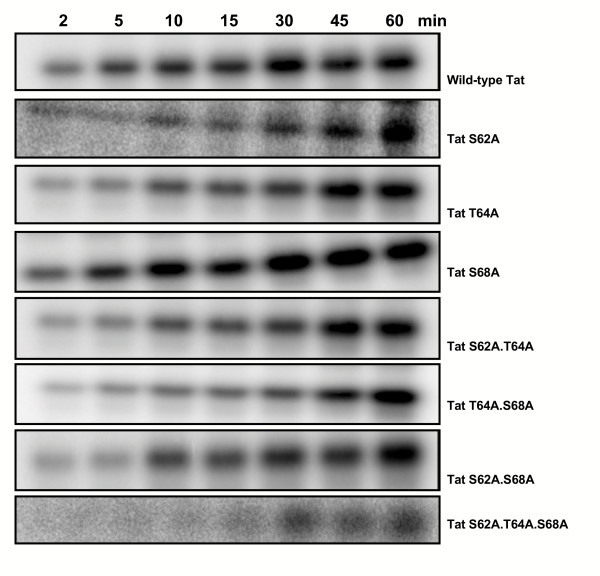
**PKR phosphorylation of HIV-1 Tat wild-type and mutants. **HIV-1 Tat wild-type and mutant proteins were expressed in BL21(DE3)pLysS cells from pET-DEST-42 expression clones, and purified by passage through a TALON™ cobalt affinity resin. PKR was immunoprecipitated from HeLa cell extracts, and activated with dsRNA in the presence of ATP. The phosphorylation reactions contained 2 μg of Tat protein, 6 μL of activated PKR suspension, and DBGA to a final volume of 12 μL. Phosphorylation was preformed at 30°C for the times indicated, in the presence of 2 μCi of γ-^32^P-ATP. Protein samples were analyzed by 15% SDS-PAGE. This figure only shows one representative gel out of three separate phosphorylation experiments performed for each protein.

**Figure 5:**
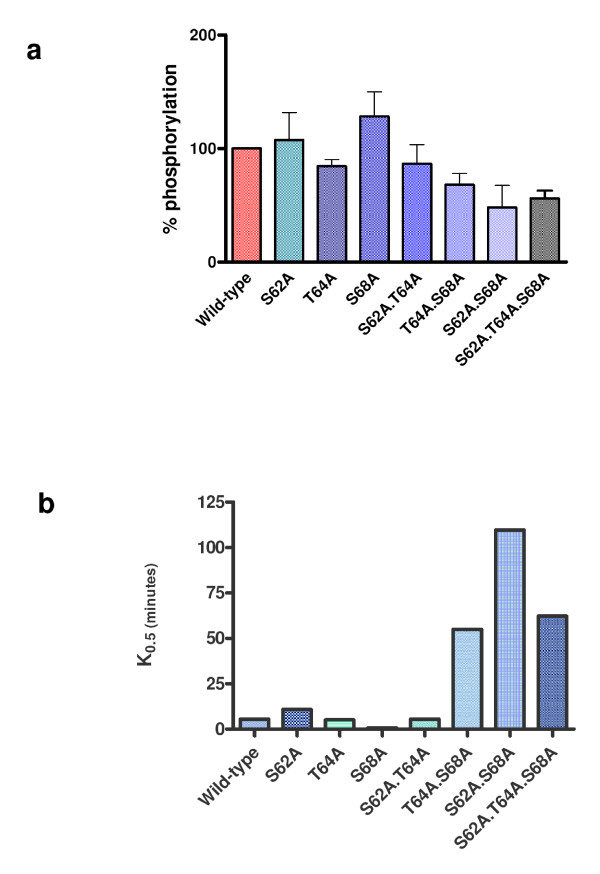
**PKR phosphorylation of HIV-1 Tat wild-type and mutants after 15 minutes and phosphorylation kinetics. (a) **Proteins were phosphorylated by activated PKR at 30°C for 15 minutes in the presence of γ-^32^P-ATP. The reaction was stopped by the addition of protein loading buffer and incubation at 4°C. Samples were analyzed by 15% SDS-PAGE. Graph shows the results for three separate experiments. **(b) **Non-linear regression analysis of PKR phosphorylation curves of wild-type and mutant proteins was performed using a one-site binding hyperbola, which describes the binding of a ligand to a receptor and follows the law of mass action. K_0.5 _is the time required to reach half-maximal phosphorylation.

Activated PKR was used to phosphorylate each of the Tat mutants as above and the reaction was allowed to proceed for 2, 5, 10, 15, 30, 45 and 60 minutes. The phosphorylated proteins were analyzed by SDS-PAGE and visualized by autoradiography (Figure 4). As can be seen from the figure, the phosphorylation of each protein by PKR varied and was the most efficient for wild-type Tat and the least efficient for the triple mutant, Tat S62A.T64A.T68A, where no sites for PKR phosphorylation were available. Scanning densitometry and non-linear regression analysis was performed and the extent of phosphorylation after 15 minutes was measured for each protein and expressed as a percentage of the wild-type protein (which is set to 100%) (Figure 5a). This time was chosen from non-linear regression analysis of the wild-type protein that indicated enzymatic phosphorylation of the wild-type protein was active at this time point. Non-linear regression analysis was performed to calculate the maximal phosphorylation for each protein (P_max_), and the time required to reach half-maximal phosphorylation (K_0.5_) (Figure 5b).

Phosphorylation of the single mutants was rapid and specific with maximal phosphorylation values (P_max_) for S62, T64 and T68 of 98.6%, 87.5% and 81.6% respectively compared to the wild type (P_max _= 82.8%) and K_0.5 _values of 10.9 min, 5.2 min and 0.8 min (wild-type = 5.5 min). This observation was also applicable to the Tat S62A.T64A mutant, which exhibited 87% phosphorylation (Figure 5a) (P_max _= 82.1%, K_0.5 _= 5.5 min). However, the percentage of phosphorylation at 15 minutes for the other double mutants and for the triple mutant decreased to 68% for Tat T64A.S68A, 48% for Tat S62A.S68A, and 56% for Tat S62A.T64A.S68A. These values also correlated well with the higher P_max _values (172.8%, 256.8% and 189.7% respectively) and K_0.5 _values (54.9 min, 109.7 min and 62.2 min respectively) for each mutant, indicating slower, less efficient and non-specific phosphorylation.

### The phosphorylation of HIV-1 Tat by PKR enhances viral transcription

**Figure 6:**
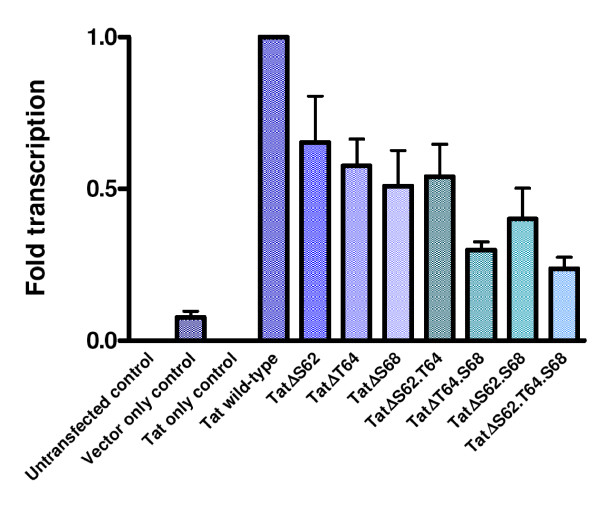
**Transactivation of the HIV-1 LTR by HIV-1 Tat wild-type and mutants. **Duplicate wells of confluent HeLa cells were transfected for 6 h with pcDNA3.2-DEST-Tat, pHIV-LTR-CAT and β-actin luciferase. Cells were harvested 24 h post transfection and assayed for CAT activity, luciferase activity and protein concentration. The graph shows the results of three separate experiments.

To examine the effect of Tat phosphorylation on its transactivation ability mammalian expression constructs containing the Tat mutants were prepared and transfected into HeLa cells. To measure Tat-specific transcription, we co-transfected with pHIV-LTR-CAT as well as with β-actin-luciferase to normalize for transfection efficiency. The transfection reaction was optimized for DNA concentration, transfection reagent concentration, and time. The results for three separate transfections are shown in Figure 6 and expressed as percentage of wild-type Tat. As expected, no transactivation of the HIV-1 LTR was observed in the untransfected control or in the absence of pHIV-LTR-CAT, and basal transcription was present at low levels (0.08-fold) in the absence of Tat. We observed significant decreases in transactivation with mutant Tat, even when a single phosphorylation site was mutated. There was a general trend to low activity as more mutations were introduced. Thus, the average transactivation by the single mutants, Tat 62A, T64A and S68A, was 58%, transactivation by the double mutants, Tat S62A.T64A, T64A.S68A and S62A.S68A, was 41%, while the triple mutant, Tat S62A.T64A.S68A, exhibited only 24% transactivation.

The differences in LTR activation observed for the individual single mutants were not large, indicating that the absence of any one of these phosphorylation residues reduced the ability of Tat to activate the HIV-1 LTR but that no single residue was more important than the other. As in the phosphorylation data, Tat S62A.T64A behaved similarly to the single mutants. The mutations that had the greatest effect were the T64A.S68A, S62A.S68A, and the triple mutant. Of the three residue combinations, the absence of T64 and S68 together had the greatest negative effect on transactivation, inducing a 3-fold decrease, which was comparable to that observed for the triple mutant (4-fold).

The absence of S62 in combination with S68 also had a marked effect on transactivation, reducing it 2.5-fold. On the other hand, the absence of S62 in combination with T64 reduced transactivation 1.8-fold. This suggests that the absence of S62 and T64 either singly or in combination is not as important for Tat-mediated transactivation as when these residues are absent in combination with S68, and may indicate a more important role for S68 in Tat transactivation. These data correlate with observations previously obtained in PKR phosphorylation experiments with these Tat mutants.

## Discussion

HIV-1 inhibits the antiviral effects of IFN by the direct binding of its Tat protein to PKR [21]. In the infected cell, Tat blocks the inhibition of protein synthesis by PKR, thus allowing viral replication to proceed. As a consequence of this interaction, Tat becomes phosphorylated at S62, T64 and S68 [22]. Here we have examined the consequences of this phosphorylation on Tat function and have shown that it results in increased and stronger binding of Tat to TAR RNA. Tat protein is an essential regulatory protein during viral transcription and binds to the positive elongation factor B (P-TEFb), through its cyclin T1 subunit, and to TAR RNA to ensure elongation of viral transcripts [31]. Since protein phosphorylation is a well-known regulatory mechanism for the control of transcription by a number of eukaryotic and viral proteins, and since phosphorylation of Rev, the other major regulatory protein of HIV-1, increases its ability to bind to RNA [32], it was important to determine if phosphorylation of Tat also resulted in the modification of its function.

The binding of Tat and TAR RNA is a necessary step for Tat to mediate viral transcription elongation [33-35]. In electrophoretic mobility shift assays, we show that Tat-P bound more TAR RNA than Tat-N, and the Tat-P-TAR complex was more resistant to competition by excess unlabelled TAR RNA. Moreover, when the NaCl concentration in the binding buffer reached 1000 mM, the dissociation of the Tat-N-TAR complex was approximately 5 times greater than that of the Tat-P-TAR complex. Together, these observations appear to indicate faster, greater and stronger binding of Tat to TAR RNA after phosphorylation by PKR. Interestingly, phosphorylated HIV-1 Rev protein has been shown to bind RNA seven times more strongly than non-phosphorylated protein, and the non-phosphorylated Rev-RNA complex dissociates 1.6 times more rapidly than the phosphorylated complex [32].

However, the precise mechanism by which phosphorylated Tat accomplishes this remains to be elucidated. It may be that the phosphorylation of Tat changes its secondary structure. This may result in an increased net positive charge by either exposing basic amino acids or masking negative amino acids, and this increases the attraction to negatively charged RNA, as in the case of cAMP response element binding protein (CREB) phosphorylation by protein kinase A and glycogen synthase kinase-3 [36]. On the other hand, phosphorylation of Tat may change the conformation of the adjacent RNA-binding domain of Tat, as observed with the phosphorylation of proteins such as HIV-1 Rev [32] and serum response factor (SRF) [37].

We examined the effect of phosphorylation on Tat-mediated transactivation of the HIV-1 LTR *in vivo *with a series of mammalian expression constructs carrying the wild-type *tat *gene or mutants of the gene with alanine substitutions at one, two, or all three of the serine/threonine PKR phosphorylation sites. Firstly, we investigated the *in vitro *phosphorylation of Tat by PKR using Tat proteins expressed and purified from analogous bacterial expression constructs. These were subject to different levels of phosphorylation by PKR and displayed distinct kinetic behaviour. Nonlinear regression analysis of the proteins indicated that PKR could not phosphorylate S62 or T64 alone in the absence of S68. These results suggest a cooperative role for the phosphorylation of S68 in conjunction with S62 and T64, although the mechanism involved and the reason for cooperation require further investigation. Overall, a gradual reduction in phosphorylation was observed as the number of mutated phosphorylation sites increased, and any phosphorylation observed with the triple mutant was shown to be non-specific, thus confirming previous published results identifying S62, T64 and S68 as the only PKR phosphorylation sites [22]. However, these findings do not exclude the possibility that there could be other sites within Tat that could be subject to phosphorylation by other kinases.

Co-transfection experiments with the mammalian expression constructs showed a 4-fold decrease in LTR transactivation with the Tat triple mutant which could not be phosphorylated by PKR. A gradual reduction in transactivation was observed as the number of mutated phosphorylation sites increased – a 2-fold reduction with the removal of one site, and 2.5-fold with the removal of two sites. Furthermore, the transfection data also suggested that the presence of S68 is necessary for optimal Tat-mediated transactivation, since its absence in conjunction with one or both of the other residues yielded the lowest levels of transcription. These results were in agreement with the *in vitro *phosphorylation data and support the hypothesis that phosphorylation of Tat may be important for its function in HIV-1 LTR transactivation.

It is relevant to note that even in the absence of all three PKR phosphorylation sites the level of transcription was still 3-fold above baseline. This may imply that Tat can still transactivate in the absence of PKR phosphorylation, although at much reduced efficiency, and/or that the protein may be phosphorylated by other kinases at other sites, for example, PKC which phosphorylates Tat at S46 [38]. Alternatively, it may be that phosphorylation could be progressive between PKR and one or more other kinases as in the case of CREB protein [36]. Furthermore, the identification of a phosphatase in enhanced Tat-mediated transactivation [39] could point to a possible, finely tuned interplay and balance between kinases and phosphatases in Tat-mediated HIV-1 transcription.

The mechanism by which the absence or presence of phosphorylation affects transactivation still requires further investigation. It could be that the introduction of an increasing number of mutations in the region 62–68 which lies next to the nuclear localization signal (aa 49–58) leads to conformational changes that prevent the protein from entering the nucleus. However, HIV-1 subtype C viruses which are rapidly expanding, carry mutations in Tat R57S and G63Q within and close to the basic domain, and yet exhibit increased transcriptional activity [40]. On the other hand, the phosphorylation of serines and threonines may facilitate the rapid folding and conformation of the protein necessary for full function as in the case of HIV-1 Rev [32]. Rev from the less pathogenic HIV-2 contains alanines in place of the serines required for phosphorylation [41,42]. It is possible to envisage a similar situation for Tat, where phosphorylation of the protein by PKR and possibly by other kinase(s) may also lead to rapid folding and changes in conformation. These changes may allow it to bind to more TAR RNA, more strongly, which in turn may lead to the formation of a stronger and more stable Tat-TAR-P-TEFb complex ensuring hyperphosphorylation of the RNAPII CTD and subsequent, successful viral transcript elongation.

## Conclusion

Overall, these results suggest that the phosphorylation of Tat by PKR plays a key role in the ability of Tat to transactivate the HIV-1 LTR, allowing the virus to use the natural antiviral responses mediated by interferon to further its own replication. This may, in part, explain the observation of increasing IFN levels in patients with advanced AIDS. The gradual reduction in transactivation observed with the decreasing absence of phosphorylation residues suggest that the presence of all PKR phosphorylation sites within the protein may be required for the optimal function of Tat in transactivation, and that the absence of S68, especially when in combination with T64, has a greater negative impact on transactivation.

## Methods

### Plasmids and proteins

The plasmid, pTZ18-TAR80 was a kind gift from Dr. E. Blair, and was used for *in vitro *transcription of TAR RNA after digestion with *Hin*D III. A β-actin luciferase reporter gene plasmid was used as a transfection control to normalize transfection efficiency and was provided by Assoc. Prof. Nick Saunders, CICR, University of Queensland, Brisbane. The pHIV-LTR-CAT construct used in transfection experiments, the destination vector, pET-DEST42 (Invitrogen, CA, USA), and the pET-DEST42-Tat86 construct were a gift from Dr. David Harrich, QIMR, Brisbane. The mammalian expression vector, pcDNA3.2-DEST was purchased from Invitrogen (CA, USA) and was used as the destination vector for the construction of the Tat86 wild-type and mutant constructs.

Synthetic HIV-1 Tat(1–86) protein was a gift from Dr. E. Blair. The protein is a chemically synthesized, full-length HIV-1(Bru) Tat (amino acids 1–86). Histidine-tagged HIV-1 Tat86 was expressed in BL21(DE3)pLysS cells (Invitrogen, CA, USA) and purified in the laboratory of Dr. David Harrich, QIMR, Brisbane. Histidine-tagged HIV-1 Tat86 phosphorylation mutants were prepared as described elsewhere in this method.

PKR was prepared as described elsewhere in this method.

### Preparation of histidine-tagged HIV-1 Tat86 phosphorylation mutants

Bacterial expression constructs were prepared using the prokaryotic expression vector, pET-DEST42-Tat86. Mutations were introduced in the *tat *gene at the three PKR phosphorylation sites: serine 62, threonine 64 and serine 68, by site-directed mutagenesis using complementary synthetic oligonucleotide primers (Proligo, Genset Pacific, Lismore, Australia) encoding the mutation of the residue, or residues, to alanine. The reaction for site-directed mutagenesis contained 32 μL distilled water, 5 μL *Pfu *I 10X reaction buffer (Promega, USA), 100 ng pET-DEST42-Tat86, 5 μL 5' oligonucleotide primer at a concentration of 25 ng/μL, 1 μL 10 mM dNTP mix, and 3 Units *Pfu *I DNA polymerase (Promega, USA). The reaction was subjected to PCR with the following cycling conditions: 95°C for 30 seconds, 18 cycles at 95°C for 30 seconds/55°C for 1 minute/68°C for 15 minutes, hold at 4°C. Electrocompetent JM109 cells were prepared in the laboratory and transformed with 2 μL of PCR reaction. Minipreps were prepared from selected ampicillin-resistant colonies and sequenced to confirm the mutation in the construct.

Mammalian expression constructs were prepared using Gateway Cloning Technology (Invitrogen, USA) to transfer the mutated *tat *genes from pET-DEST42-Tat86 wild type and mutants to the mammalian expression vector, pcDNA3.2-DEST, according to the protocol supplied by the manufacturer.

### Expression and purification if HIS-tagged Tat mutant proteins

Competent BL21(DE3)pLysS cells (Dr. David Harrich, QIMR, Brisbane, Australia) were transformed with 1 μL of pET-DEST42-His-Tat86 wild-type or mutants, and plated. A single ampicillin resistant colony was resuspended in 10 mL of LB broth/amp and incubated overnight at 37°C. This culture was added to 500 mL of LB broth/amp and incubated in an orbital shaker, at 37°C until the OD_600 _was 0.6. The culture was inoculated with IPTG (Roche, Germany) to a final concentration of 200 μg/mL and incubation was continued for a further 2 hours. Cells were pelleted; the pellet was resuspended in 2 volumes of 6 M guanidine-HCl, pH 8.0 and incubated at room temperature overnight. The suspension was centrifuged at 14500 × *g *for 20 minutes, and the supernatant was centrifuged at 100 000 × *g *for 30 minutes. The supernatant was loaded onto a 1 mL equilibrated, packed resin (TALON™ Metal Affinity Resin, BD Biosciences Clontech, USA). To equilibrate, the resin was washed twice with 10 mL of Milli-Q water and charged by incubating with 5 mL of 0.3 M CoCl_2 _at room temperature for 5 minutes. The resin was then washed extensively with water, and equilibrated in 6 M guanidine-HCl, pH 8.0. The HIS-tagged protein was allowed to bind to the resin by incubation on a rocking platform, at room temperature, for 1 hour. The resin was then sedimented at 700 × *g *for 2 minutes, and washed with 6 M guanidine-HCl, pH 8.0 for 5 minutes. The resin was sedimented as above and washed with 6 M guanidine-HCl, pH 6.0 for 5 minutes. The resin was loaded onto an empty column (Poly-Prep ion exchange column, Bio-Rad, USA), and the wash allowed to flow through. The HIS-tagged protein was eluted with 4 mL of 6 M guanidine-HCl, pH 4.0, and collected in 500 μL fractions. Fractions were dialysed in 0.1 mM DTT in PBS, at room temperature, overnight, and then centrifuged at 14500 × *g *for 2 minutes. To identify fractions containing the HIS-tagged protein, 5–20 μL aliquots were analysed by 15% SDS-PAGE and stained with Coomassie blue. Fractions containing protein were assayed for protein concentration (Bio-Rad Protein Assay Dye Reagent Concentrate, Bio-Rad, USA), and by Western blot against a 1:1000 dilution of monoclonal anti-poly HISTIDINE Clone HIS-1 antibody (Sigma Aldrich, USA). Aliquots of fractions were stored at -80°C in 10 mM DTT in PBS.

### *In vitro *phosphorylation assays

PKR was purified from HeLa cell extracts as described previously [43]. Briefly, confluent HeLa cells in 75 cm^2 ^flasks were lysed in 1 mL of Buffer 1 (20 mM Tris, pH 7.6, 50 mM KCl, 400 mM NaCl, 1 mM EDTA, 1% Triton X-100, 20% glycerol, 200 μM PMSF, 5 mM mercaptoethanol), and centrifuged at 13500 × *g *for 30 minutes at 4°C. The supernatant was incubated in ice, for 30 minutes, with 2 μL of a 1:10 dilution of specific monoclonal antibody 71/10 (Dr. A. Hovanessian, Pasteur Institute, France), and then at 4°C overnight with 65 μL of protein G-sepharose (Amersham Biosciences, Sweden), with continuous rotation. Protein G-sepharose-PKR was sedimented, washed three times with Buffer 1, and three times with DBGA (10 mM Tris, pH 7.6, 50 mM KCl, 2 mM magnesium acetate, 20% glycerol, 7 mM β-mercaptoethanol). PKR was activated by incubating 120 μL of this suspension with 80 μL of DBGB (DBGA + 2.5 mM MnCl_2_), synthetic dsRNA (Sigma Aldrich, USA) to a final concentration of 0.5 μg/mL, and 20 μL of 2 mg/mL ATP (Sigma Aldrich, USA), at 30°C for 15 minutes.

Phosphorylation reactions for Tat proteins contained 2 μg of HIV-1 Tat, unless otherwise indicated in the figure legend, 6 μL of activated PKR suspension, and DBGA to a final volume of 12 μL. Phosphorylation was performed at 30°C for 1 hour, unless otherwise stated, in the presence of 2 μCi of γ-^32^P-ATP (Perkin-Elmer, USA). For measuring the extent of phosphorylation of the mutant Tat proteins, phosphorylation was stopped after 2, 5, 10, 15, 30, 45, and 60 minutes by the addition of protein loading buffer. Samples were analysed by 15% SDS-PAGE, and proteins were visualized by autoradiography, and scanning densitometry in a STORM 860 phosphorimager with ImageQuant^® ^software (Molecular Dynamics, USA).

### Electrophoretic mobility shift assay (EMSA)

TAR RNA was synthesized from 0.8 μg of pTZ18TAR80 using a commercial *in vitro *transcription system (MAXIscript™ T7 kit, Ambion, USA) according to the protocol supplied with the kit. HIV-1 Tat was phosphorylated (Tat-P) with activated PKR for 1 hour, as described above, or in the absence of γ-^32^P-ATP (Tat-N). Tat-P and Tat-N were allowed to equilibrate at 30°C for 10 minutes in Binding Buffer (10 mM Tris, pH 7.6, 1 mM DTT, 1 mM EDTA, 50 mM NaCl, 0.05% glycerol, 0.09 μg/μL BSA), before incubating at 30°C for 10 minutes with 2.5 × 10^5 ^cpm of ^32^P-TAR RNA. The Tat-TAR RNA complexes were separated on a 5% acrylamide/0.25X TBE gel (0.45 M Tris, 0.45 M boric acid, 0.1 M EDTA, pH 8.0), for 3–4 hours, at 10 mA, and visualized by autoradiography.

### Transfection assays

Transfections were performed in duplicate in 6-well plates. HeLa cells were diluted in Modified Eagle's Medium (Invitrogen, USA) supplemented with 10% foetal bovine serum (Trace Scientific, Melbourne, Australia), antibiotics and glutamine (Invitrogen, USA), to yield 5 × 10^5 ^cells/mL. Each well was seeded with 2 mL of this cell suspension, and incubated at 37°C/5% CO_2 _for 24 hours or until the cell monolayer was 80–90% confluent. A solution of 625 μL of serum-free medium and 10 μg of total DNA (3.3 μg β-actin-luciferase, 3.3 μg pcDNA3.2-DEST-Tat, 3.3 μg pHIV-LTR-CAT) was mixed with 600 μL of serum-free medium containing 25 μL of Lipofectamine 2000 (Invitrogen, USA), and incubated at room temperature for 20 minutes. The cells were washed twice with serum-free medium, inoculated with the DNA-Lipofectamine mixture, and incubated at 37°C for 6 hours. The DNA solution was replaced with complete medium and the cells wee incubated as above for 24 hours. The cells were harvested and assayed for CAT activity using the CAT ELISA kit (Roche, Switzerland) according to the protocol supplied with the kit, for luciferase activity using the Luciferase Assay System (Promega, USA) according to the supplied protocol, and for protein concentration (Bio-Rad Protein Assay Dye Reagent Concentrate, Bio-Rad, USA).

## Competing interests

The author(s) declare that they have no competing interests.

## Authors' contributions

LEM was responsible for the experiments described and contributed to the drafting of the manuscript. TW performed the optimization experiments for the phosphorylation of Tat by PKR. DH participated in the design of the study, provided reagents and critically read the manuscript. NAJM conceived and coordinated the study, and contributed to the drafting of the manuscript.
